# *In vitro* Natural Killer Cell Immunotherapy for Medulloblastoma

**DOI:** 10.3389/fonc.2013.00094

**Published:** 2013-04-19

**Authors:** Lucia Fernández, Raquel Portugal, Jaime Valentín, Roberto Martín, Hannah Maxwell, Marta González-Vicent, Miguel Ángel Díaz, Inmaculada de Prada, Antonio Pérez-Martínez

**Affiliations:** ^1^Department of Hemato-Oncology and Stem Cell Transplantation, Hospital Infantil Universitario Niño JesúsMadrid, Spain; ^2^Department of Pathology, Hospital Infantil Universitario Niño JesúsMadrid, Spain; ^3^School of Medicine, Cardiff UniversityCardiff, Wales, UK

**Keywords:** natural killer cells, medulloblastoma, NKG2D ligands, NKG2D receptor, HLA-I

## Abstract

How the immune system attacks medulloblastoma (MB) tumors effectively is unclear, although natural killer (NK) cells play an important role in immune defense against tumor cells. Interactions between receptors on NK cells and ligands expressed by tumor cells are critical for tumor control by immunotherapy. In this study, we analyzed tumor samples from 54 MB patients for expression of major histocompatibility complex class I-related chains A (MICA) and UL16 binding protein (ULPB-2), which are ligands for the NK group 2 member D activatory receptor (NKG2D). The percentage of MICA and ULBP-2 positive cells was higher than 25% in 68% and 6% of MB patients, respectively. A moderate-high intensity of MICA cytoplasmic staining was observed in 46% MB patients and weak ULBP-2 staining was observed in 8% MB patients. No correlation between MICA/ULBP-2 expression and patient outcome was found. We observed that HTB-186, a MB cell line, was moderately resistant to NK cell cytotoxicity *in vitro*. Blocking MICA/ULBP-2 on HTB-186, and NKG2D receptor on NK cells increased resistance to NK cell lysis *in vitro*. However, HLA class I blocking on HTB-186 and overnight incubation with IL-15 stimulated NK cells efficiently killed tumor cells *in vitro*. We conclude that although NKG2D/MICA-ULBP-2 interactions have a role in NK cell cytotoxicity against MB, high expression of HLA class I can protect MB from NK cell cytotoxicity. Even so, our *in vitro* data indicate that if NK cells are appropriately stimulated, they may have the potential to target MB *in vivo*.

## Introduction

Medulloblastoma (MB) is a highly aggressive pediatric primitive neuroectodermal tumor usually located in the posterior fossa. Current treatment for MB consists of a combination of surgical resection, systemic multidrug chemotherapy, and craniospinal radiation (Von Hoff and Rutkowski, 2012). Up to 75% of patients with average-risk MB with localized disease, who undergo complete surgical resection and are older than 3 years old, can achieve complete and persistent remission although long-term sequels may occur (Douglas et al., 2004). In contrast, in patients with high-risk MB with metastatic disease, large residual tumors, relapsing disease and of a young age have a poor prognosis (Pérez-Martínez et al., 2004; Butturini et al., 2009).

Knowledge of the molecular characteristics of MB has increased during recent years (Leary and Olson, 2012). An understanding of the growth control mechanisms involved in MB development has allowed a better classification and development of target therapies (Huse and Holland, 2010). Many signaling pathways have been identified and numerous anticancer drugs have been designed to target specific proteins in these pathways. However, minimal antitumor activity has been reported (Fouladi et al., 2007; Rossi et al., 2008). Although numerous novel immunotherapy approaches are being explored, the dysfunction of patients T cells and the lack of tumor-specific targets for cytotoxic T lymphocytes have limited specific immune cell therapies (Sonabend et al., 2012). Although there has not been any clear clinical success (Salmaggi et al., 1994; Silvani et al., 1994; Sankhla et al., 1996), recent *in vitro* studies have demonstrated that natural killer (NK) cells are able to lyse MB cell lines (Castriconi et al., 2007). Because NK cells play a major role in the immune defense against tumors, they are good candidates for new immunotherapeutic approaches (Geller and Miller, 2011). The NK cell antitumor effect is controlled through the balance of signals mediated by activating and inhibitory receptors found on each NK cell. Tumor transformation down-regulates HLA class I expression, ligands to NK inhibitory receptors, and up-regulates ligands for NK activating receptors, and results in NK cell-mediated tumor lysis. MB cell lines express specific ligands that trigger NK activating receptors and thus are susceptible to NK-mediated cytotoxicity (Castriconi et al., 2007). Major histocompatibility complex class I-related chain A (MICA) and UL16 binding protein 2 (ULBP-2) are tumor cell surface ligands that bind NK cell activating receptor NKG2D, and are prevalent in malignant brain tumors (Friese et al., 2003; Castriconi et al., 2007; Geller and Miller, 2011). Interactions between NKG2D receptors on the surface of NK cells and their ligands (NKG2DL) on tumor cells has been proposed as critical for NK cell cytotoxicity against tumor cell lines, but also primary tumors (Friese et al., 2003; Kloess et al., 2010; Bae et al., 2012; Pérez-Martínez et al., 2012).

In this study, we show high expression of MICA on primary MB cells and the important role of NKG2D/NKG2DL for NK cell cytotoxicity using HTB-186, a MB cell line. However, high expression of HLA class I on MB cells cause them to become resistant to NK cytotoxicity. We also demonstrate that blocking HLA class I on MB cells and/or IL-15 stimulated NK cells can overcome the inhibitory effect mediated by HLA class I overexpression on tumor cells.

## Materials and Methods

Our local Ethics Committee approved this study protocol and all patients’ guardians gave their informed consent to this study.

### Patients and clinical care and evaluation

Between 1990 and 2010, 54 MB patients were diagnosed and treated at our institution (Table 1). Mean age at diagnosis was 6.5 ± 4.2 years (26% patients were younger than 3 years old). Gender percentages were 59% male and 41% female. MB histological analysis demonstrated the following classifications: 74% classical, 22% nodular, and 4% anaplastic. Metastasis at diagnosis was present in 32% of patients. Neurosurgery complete debulking was performed in 47% patients. Radiotherapy was performed in 82% of the patients. High-dose chemotherapy and autologous stem cell rescue was performed in 33% of patients. MB relapses occurred in 37% of patients.

**Table 1:** **Patient characteristics**.

	*n*	%	Overall survival	*P*
Patient numbers (1990–2010)	54			
Patient numbers (1990–2000/2001–2010)	24/30	44/54		ns
Age (years)	6.5 ± 4.2			
Age (<3/>3 years)	14/40	26/74	42 ± 1/53 ± 1	ns
Sex (male/female)	32/22	59/41	54 ± 1/43 ± 1	ns
Histology (classic/nodular/anaplastic)	39/11/2	74/22/4	46 ± 1/68 ± 1/50 ± 3	ns
Resection: CR/PR	25/28	47/53	62 ± 1/40 ± 1	0.05
Metastasis (no/yes)	34/16	68/32	56 ± 1/30 ± 1	0.05
Radiotherapy (yes/no)	40/9	82/18	60 ± 1/11 ± 1	0.000*
Relapse (yes/no)	18/31	37/63	13 ± 1/69 ± 1	0.001*
HDCT (yes/no)	15/31	33/67	29 ± 1/57 ± 1	ns
Status (CR/PR/dead)	22/4/22	46/8/46		
Follow up (months)	60.0 ± 2.6			
Survival (1990–2000/2001–2010)			50 ± 7 (52 ± 1/40 ± 1)	ns

Data are expressed as mean ± SEM. NS: no significance; CR: complete remission, PR: partial remission; HDCT: high dose chemotherapy and autologous stem cell rescue.*Significance at multivariate analysis.

### Antibodies and flow cytometry analysis

The following fluorochrome-labeled monoclonal antibodies (mAbs) against human antigens were obtained from R&D Systems (Minneapolis, MN, USA): MICA-PE, ULBP-1-PE, ULBP-2-APC, ULBP-3-PE, and ULBP-4-PerCp-Cy5. Fluorochrome-labeled mAbs against MICA/MICB were obtained from Biolegend Inc. (San Diego, CA, USA). Fluorochrome-labeled mAb against HLA-ABC-PE was obtained from Becton Dickinson, Franklin Lakes, NJ, USA. Mean fluorescent intensity for MICAB, MICA, ULBP-1, ULBP-2, ULBP-3, and ULBP-4 was determined in NB1691, K562, HTB-185, HTB-186, and HTB-187 cell lines by multiparametric flow cytometry (Becton Dickinson, FACSCanto II).

The mouse IgG2A isotype antibody and mouse anti-human IgG2A MICA monoclonal antibody were purchased from R&D Systems. The anti-HLA class I antibody used in blocking experiments, W6/32 IgG2a, was kindly provided by Dr. S. Stevanovic (Tubingen University, Germany). The mouse IgG2A anti-human ULPB2 monoclonal antibody and anti-NKG2D IgG1 antibody were purchased from Abcam (Cambridge, UK). All mAbs were used at a final concentration of 10 μg/ml, except for anti-HLA class I that we used 20 μg/ml.

### Immunohistochemistry

Specimens of pediatric tumors from 54 children with MB were included on tissue microarray and analyzed as previously described (Pérez-Martínez et al., 2012). Briefly, 3 μm tissue sections were paraffin-embedded for 30 min then put into a PT Link (Dako) to conduct the pre-treatment processes of deparaffinization, rehydration, and epitope retrieval for 2 h. After that, the slides were loaded on to an Autostainer (Dako) where the tissue was blocked for endogenous peroxidase and stained using antibodies against MICA (1:10, R&D Systems) and ULBP-2 (1:50, R&D Systems). The Envision Flex (secondary antibody) and immunodetection system diaminobenzidine was used. The level of MICA and ULBP-2 expression was assessed by the intensity of cytoplasmic staining as follows: (0 none, 1+ weak, 2+ moderate, and 3+ strong) and the percentage of positive cells (<25, 25–50, 50–75, and >75%). Pancreas carcinoma served as a positive control for ULBP-2 (Chen et al., 2008) and normal breast epithelium as a positive control for MICA (Chang et al., 2011).

### Cytotoxicity assays and cell lines

The cytotoxicity of NK cells was monitored using a conventional 2-h europium-TDA release assay (Perkin-Elmer Wallac, Turku, Finland) as described previously (Blomberg et al., 1995). Fresh peripheral blood mononuclear cells (PBMCs) obtained from healthy volunteers were used as effector cells. K562 (erythroleukemia cell line, ATCC), HTB-186 (a MB cell line kindly provided by Dr. P. Sánchez Gómez, Instituto de Salud Carlos III, Madrid, Spain), and NB1691 (neuroblastoma cell line, kindly provided by Dr. A. Davidoff, St. Jude’s Children’s Research Hospital, Memphis, TN, USA) were used as the target cells. In brief, target cells were labeled with a fluorescence-enhancing ligand (BATDA). This hydrophobic ligand quickly penetrates the cell membrane. Within the cell, hydrolysis of ester bonds results in the ligand becoming hydrophilic and therefore unable to pass through the cell membrane. Cytolysis, however, results in the release of the ligand and ultimately a reaction of the ligand with the europium to form a stable, fluorescing chelate, which was evaluated fluorometrically (Infinite F200 reader TECAN Group Ltd., Männedorf, Switzerland). The number of NK cells was calculated by multiplying the lymphocyte counts to the percentage of CD3^−^CD56^+^ NK cells. The following formulas were used to calculate spontaneous and specific cytotoxicity:

$$\begin{array}{l} \%\text{ Specific release}=(\text{experimental release}-\text{spontaneous release})\\ \;/(\text{maximumrelease }-\text{ spontaneous release})\times 100.\\ \;\%\text{ Spontaneous release}=(\text{spontaneous release }-\text{ background})\\ \;/(\text{maximumrelease}-\text{background})\times 100. \end{array}$$

### IL-15 stimulated NK cells and antibody blocking experiments

Fresh PBMCs from healthy controls were stimulated overnight with 25 ng/ml IL-15 (R&D Systems). Cultures were grown in complete culture medium (RPMI 1640 supplemented with 10% of heat-inactivated fetal bovine serum, 100 IU/ml penicillin, 100 ng/ml streptomycin, and 2 mM/l-glutamine) in a humidified atmosphere of 5% CO_2_ and 95% air.

HTB-186 cell line was incubated with mouse IgG2A isotype antibody, mouse anti-human MICA monoclonal IgG2A antibody, anti-HLA class I IgG2A antibody, and mouse anti-ULPB-2 monoclonal IgG2A antibody for 2 h at 37°C. PBMCs were incubated with NKG2D IgG1 antibody for 30 min at room temperature, and then washed twice in fresh complete medium. Following this, cytotoxicity was measured as described above.

### Statistical analysis

Data was analyzed during August 2012. Results are given as means ± standard error, unless otherwise indicated. Significance levels were determined by Student *t*-test analysis. *P* values of 0.05 or less were considered significant. Overall survival (OS) was analyzed using Kaplan–Meier test and the log-rank test for univariate analysis. The correlation between the magnitude of the increase in NK cell cytotoxicity and NKG2DL expression on tumor cell lines was determined by Pearson’s method, setting statistical significance at *P* < 0.05.

## Results

### Patients and outcome

Between 1990 and 2010, 54 MB patients were diagnosed and treated at our institution (Table 1). With a median of 60 months, OS was 50 ± 7%. Univariate analysis demonstrated that quality of resection, metastasis at diagnosis, radiotherapy, and relapse impacted on OS. Multivariate analysis showed radiotherapy and relapses impacted on OS.

### Immunohistochemistry

Of the MB patients, 46% demonstrated strong positive MICA expression (38% moderate and 8% high) (Table 2). Weak expression was observed in 42% MB patients and 12% showed no MICA positive staining. A total of 92 and 8% MB patients had either none or weak ULBP-2 positive staining, respectively. A total of 62% MB patients showed extensive expression of MICA, and of these 24% had 50–75% MICA positive cells and 38% had >75% MICA positive cells. A total of 38% MB patients showed low expression of MICA, where 6% had 25–50% MICA positive cells and 32% had <25% MICA positive cells.

**Table 2:** **MICA/ULBP-2 positive cells and intensity of cytoplasmic staining by immunohistochemistry on medulloblastoma tumors**.

	Positive cells *n* (%)			
	<25	25–50	50–75	>75
MICA	16 (32)	3 (6)	12 (24)	19 (38)
ULBP-2	47 (94)	3 (6)	0 (0)	0 (0)

	**Intensity of cytoplasmic staining *n* (%)**			
	**None**	**Weak**	**Moderate**	**High**

MICA	6 (12)	21 (42)	19 (38)	4 (8)
ULBP-2	46 (92)	4 (8)	0 (0)	0 (0)

**Figure 1:**
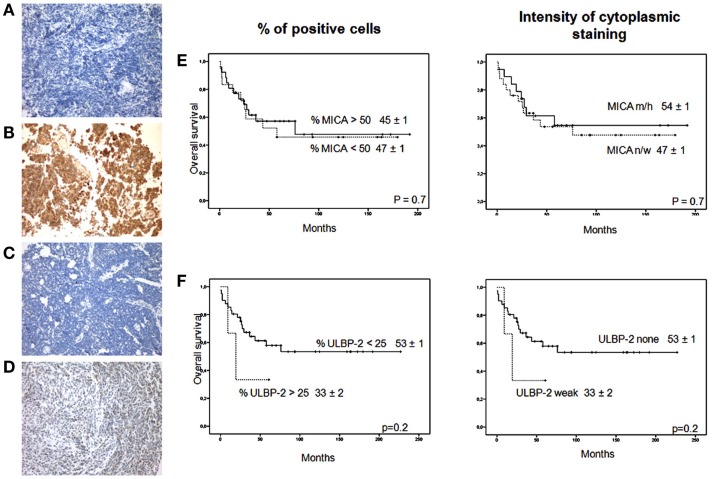
**MICA and ULBP-2 staining in MB**. **(A)** MICA negative staining in a sample of MB; **(B)** MICA high positive staining in a sample of MB; **(C)** ULBP-2 negative staining in a sample of MB; **(D)** ULBP-2 weak staining in a sample of MB. Magnification × 200 for all samples. Impact of **(E)** MICA expression (% > 50%, *n* = 31 vs. % < 50%, *n* = 19 and intensity cytoplasmic staining, none/weak, *n* = 27, vs. moderate/high, *n* = 23); and **(F)** ULPB-2 expression (% > 25%, *n* = 3 vs. % < 25%, *n* = 47 and intensity of cytoplasmic staining, none, *n* = 46, vs. weak, *n* = 4) for overall survival. n/w, none/weak; m/h, moderate/high.

The percentage of ULPB-2 immunoreactivity in most MB patients was <25%. Values between 25–50% were present in 6% of patients (Figures 1A–D). MICA and ULBP-2 expression did not have an impact on OS (Figures 1E,F). Thus, approximately 50% of the samples had high MICA expression on a high percentage of tumor cells and most samples had no ULBP-2 expression.

### Surface expression of NKG2DLs on tumor cell lines

**Figure 2:**
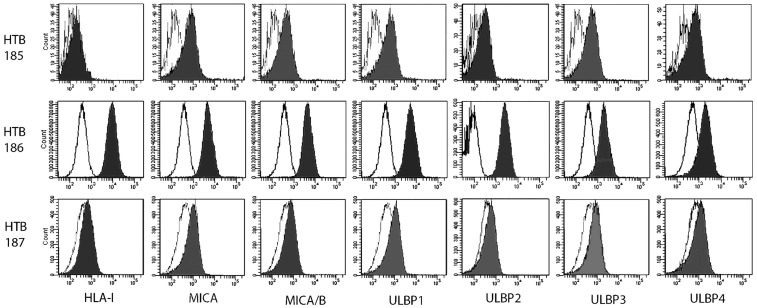
**Medulloblastoma cell lines, (HTB-185, HTB-186 MB, HTB-187), mean fluorescence intensity (MFI) was determined by multiparametric flow cytometry (Becton Dickinson, FACSCanto II) for HLA-I expression and ligands for the NKG2D activating receptor**.

Cell surface expression of NKG2DLs on tumor cell lines was assessed by FACS analysis. Mean fluorescence intensity ratio was determined by the fold-increase over isotype control using mouse mAbs. The HTB-186 tumor cell line expressed the highest levels of HLA class I, MICA, ULBP-2, ULBP-3 and the lowest NKG2DLs/HLA class I ratio (Table 3 and Figure 2).

**Table 3:** **Cell surface expression of HLA-I and NKG2D ligands and NKG2D ligands/HLA-I ratio on medulloblastoma cell lines (HTB-185, HTB-186, HTB-187), neuroblastoma cell line (NB1691) and erythroleukaemia cell line (K562) measured by mean fluorescence intensity (MFI)**.

Ligands	HTB-185	HTB-186	HTB-187	NB1691	K562	Ligands	HTB-185	HTB-186	HTB-187	NB1691	K562
HLA-I	1.50	28.57	1.34	1.83	1.88	MICA/HLA-I	2.21	0.52	1.12	3.50	6.07
MICA	5.66	14.96	1.98	6.39	11.44	MICAB/HLA-I	3.78	0.50	1.48	2.50	7.29
MICAB	3.31	14.23	1.49	4.57	13.75	ULBP-1/HLA-I	3.15	0.41	1.57	4.44	7.51
ULBP-1	4.73	11.72	2.10	8.11	14.16	ULBP-2/HLA-I	1.98	1.13	1.03	4.95	8.54
ULBP-2	2.97	32.42	1.37	9.04	16.10	ULBP-3/HLA-I	2.89	0.36	1.28	2.17	2.58
ULBP-3	4.33	10.37	1.72	3.96	4.85	ULBP-4/HLA-I	1.82	0.12	1.07	2.00	2.84
ULBP-4	2.73	3.34	1.43	3.65	5.34						

### NK cell cytotoxicity and blocking experiments

**Figure 3:**
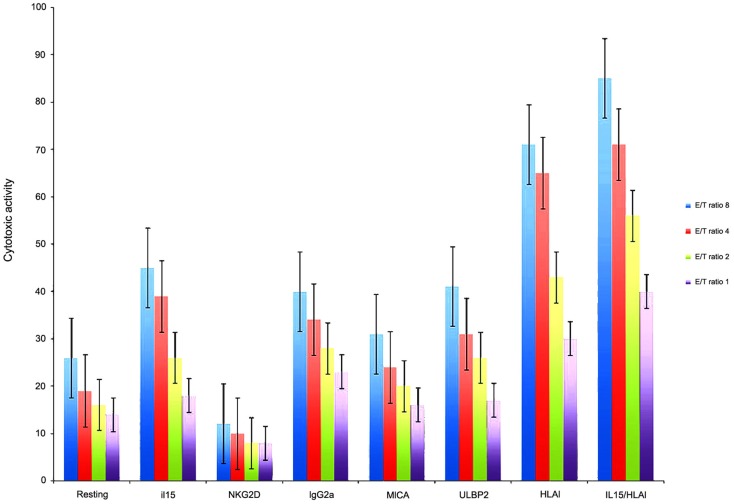
**HTB-186 MB cell line susceptibility to: resting NK cells, IL-15 stimulated NK cells, NKG2D, MICA, ULBP-2 and HLA class I blocking experiments at different effector target (E/T) ratio**. Blocking MICA, ULBP-2 on HTB-186 cells and NKG2D on NK cells reduced NK cell mediated cytotoxicity. HLA class I blocking on HTB-186 cells and IL-15 stimulated NK cells increased NK cell mediated cytotoxicity.

Because from MB cell lines, HTB-186 expressed the highest levels of ligands for NK cell receptors, we chose it from MB cell lines, for cytotoxicity experiments. We observed HTB-186 cell line was the most resistant cell line to resting NK cell cytotoxicity (26, 19, 16, 10% vs. 50, 34, 32, 31% on K562 and 42, 29, 19, 14% in NB1691 at ratio 8/4/2/1:1, respectively). To investigate the importance of NKG2D-MICA/ULBP-2 interactions in MB patients we examined *in vitro* NK cytotoxicity after blocking NKG2D receptor on NK cells and blocking MICA and ULBP-2 on HTB-186 cell line. NKG2D blocking decreased NK cell cytotoxicity twofold and blocking MICA and ULBP-2 decreased cytotoxicity 1.5- and 1.2-fold when compared with groups using resting NK cells and IgG2A, respectively. IL-15 stimulated NK cells increased cytotoxicity twofold when compared with resting NK cells, however the effect was similar to observed by IgG2A control. Moreover, blocking HLA class I on tumor cells increased NK cell cytotoxicity 3.4- and 2-fold when compared with groups using resting NK cells and IgG2A, respectively. Blocking HLA class I on tumor cells and IL-15 stimulated NK cells increased cytotoxicity 3.7- and 2.1-fold when compared with groups using resting NK cells and IgG2A, respectively (Figure 3).

### NK cell cytotoxicity and blocking experiments and surface expression of NKG2DL/HLA class I ratio on tumor cell lines

A positive correlation analysis between MICA/HLA class I, ULBP-1/HLA class I and ULBP-4/HLA class I expression and resting NK cell cytotoxicity was found (Pearson > 98% and *P* < 0.05). In addition, a positive correlation between MICB/HLA class I expression and IL-15 stimulated NK cells was observed (Pearson > 99% and *P* < 0.05). Finally, a positive correlation between ULBP-1/HLA class I, ULBP-3/HLA class I and ULBP-4/HLA class I expression and NKG2D blocking NK cells was found (Pearson > 99% and *P* < 0.05). No significant correlations were found when we compared NK cytotoxicity and HLA class I expression or NKG2L alone. These findings suggest that the NKG2DL/HLA class I ratio predicts NK cell cytotoxicity better than NKG2DL expression alone.

## Discussion

This study investigated the potential role of NK cell immunotherapy for MB based on the engagement of NKG2D receptor by MICA/ULBP-2 ligands. We demonstrated that NKG2DLs, especially MICA, expressed by MB primary tumors and ULPB-2 on HTB-186 cell line, were overexpressed. Despite MICA and ULBP-2 overexpression, NK cell cytotoxicity was not as high as we expected when NK cells were incubated with HTB-186. However, we observed a decrease in NK cell cytotoxicity when we blocked NKG2D/MICA-ULBP-2 interactions. This suggests that although NK cell cytotoxicity against MB is low, it is maintained by NKG2D/MICA-ULBP-2 interactions. In addition, high HLA class I expression on MB cells blocked NK cell cytotoxicity mediated by NKG2DL, and blocking HLA class I on HTB-186 cells increased NK cell cytotoxicity. These data are in agreement with results reported in glioma cell lines (Friese et al., 2003). However, our data contrast with the high susceptibility of other MB cell lines to NK cell cytotoxicity and the suggested major role of DNAM-1 and NKG2D receptors (Castriconi et al., 2007). Although NK cell triggering mediated by NKG2D-NKG2DL interactions have been proposed as critical for NK cell cytotoxicity against most pediatric malignancies we hypothesized a determinant role for the inhibitory signals derived from HLA class I expression on tumor cells. However, HLA class I expression in many cancers and MB is heterogeneous, reflecting the status of the original cell before transformation (Smith et al., 2011). Malignant transformation may be associated with abnormalities in HLA class I expression and in major histocompatibility complex class I-related molecules (Raffaghello et al., 2007). Consequently, we observed that susceptibility to NK cell cytotoxicity depends on NKGD2L/HLA class I expression, and higher or lower ratios correspond to higher or lower NK cytotoxicity.

High expression of NKGD2Ls has been described for other brain malignant tumors. However, in parallel, they also had increased HLA class I expression (Friese et al., 2003), which may allow tumor cells escape NK cell immunosurveillance. Furthermore, the high expression of HLA class I may contribute to a more malignant phenotype of MB by activation of ERK1/2 and result in a poor prognosis (Raffaghello et al., 2007; Smith et al., 2009; Smith et al., 2011). Because the brain lacks specific brain-associated lymphoid tissue, tumor antigens elicit transient innate inflammatory immune responses, such as those mediated by NK cells, but no adaptive immunity (Smith et al., 2011).

Interestingly, NK cell stimulation with IL-15 circumvented the ability of HLA class I to inhibit NK cell cytotoxicity compared with resting NK cells. Thus, *ex vivo* IL-15 stimulated NK cells could be a strategy for treatment of MB patients, as has been reported for other pediatric solid tumors (Cho et al., 2010). Although MB survival has improved, a total cure is still not available. In this study, average-risk patient had 55% chance of survival. However, metastatic disease at diagnosis, incomplete resections, and a younger age at diagnosis had <40% chance survival. Furthermore, MB relapse patients have a lower chance (<15%) to be successfully treated. Thus, new strategies for the treatment of MB should be developed.

In summary, this study provides fundamental insights into the crosstalk between NK cells and MB tumors based on receptor-ligand interactions. NK cells may play an important role in cytotoxicity of MB tumors and may therefore be a new target for future medical therapies, especially in high-risk MB patients.

## Conflict of Interest Statement

The authors declare that the research was conducted in the absence of any commercial or financial relationships that could be construed as a potential conflict of interest.

## References

[B1] Bae D. S. Hwang Y. K. Lee J. K. (2012). Importance of NKG2D-NKG2D ligands interaction for cytolytic activity of natural killer cell. Cell. Immunol. 276, 122–127 10.1016/j.cellimm.2012.04.011 22613008

[B2] Blomberg K. Hautala R. Lovgren J. Mukkala V. M. Lindqvist C. Akerman K. (1995). Time-resolved fluorometric assay for natural killer activity using target cells labeled with a fluorescence enhancing ligand. J. Immunol. Methods 193, 199–206 10.1016/0022-1759(96)00063-4 8699033

[B3] Butturini A. M. Jacob M. Aguajo J. Vander-Walde N. A. Villablanca J. Jubran R. (2009). High-dose chemotherapy and autologous hematopoietic progenitor cell rescue in children with recurrent medulloblastoma and supratentorial primitive neuroectodermal tumors: the impact of prior radiotherapy on outcome. Cancer 115, 2956–2963 10.1002/cncr.24341 19402050

[B4] Castriconi R. Dondero A. Negri F. Bellora F. Nozza P. Carnemolla B. (2007). Both CD133+ and CD133- medulloblastoma cell lines express ligands for triggering NK receptors and are susceptible to NK-mediated cytotoxicity. Eur. J. Immunol. 37, 3190–3196 10.1002/eji.200737546 17918205

[B5] Chang Y. T. Wu C. C. Shyr Y. M. Chen T. C. Hwang T. L. Yeh T. S. (2011). Secretome-based identification of ULBP2 as a novel serum marker for pancreatic cancer detection. PLoS ONE 6:e20029 10.1371/journal.pone.0020029 21625447 PMC3098863

[B6] Chen X. M. Xu X. Q. Sun K. Hallett W. H. Zhao J. D. Zhang D. L. (2008). NKG2D ligands expression and NKG2D-mediated cytotoxicity in human laryngeal squamous carcinoma cells. Scand. J. Immunol. 67, 441–447 10.1111/j.1365-3083.2008.02086.x 18312485

[B7] Cho D. Shook D. R. Shimasaki N. Chang Y. H. Fujisaki H. Campana D. (2010). Cytotoxicity of activated natural killer cells against pediatric solid tumors. Clin. Cancer Res. 16, 3901–3909 10.1158/1078-0432.CCR-09-3022 20542985 PMC3168562

[B8] Douglas J. G. Barker J. L. Ellenbogen R. G. Geyer J. R. (2004). Concurrent chemotherapy and reduced dose cranial spinal irradiation followed by conformal posterior fossa tumor bed boost for average-risk medulloblastoma: efficacy and patterns of failure. Int. J. Radiat. Oncol. Biol. Phys. 58, 1161–1164 10.1016/j.ijrobp.2003.09.010 15001259

[B9] Fouladi M. Nicholson H. S. Zhou T. Laningham F. Helton K. J. Holmes E. (2007). A phase II study of the farnesyl transferase inhibitor, tipifarnib, in children with recurrent or progressive high-grade glioma, medulloblastoma/primitive neuroectodermal tumor, or brainstem glioma: a children’s oncology group study. Cancer 110, 2535–2541 10.1002/cncr.23078 17932894

[B10] Friese M. A. Platten M. Lutz S. Z. Naumann U. Aulwurm S. Bischof F. (2003). MICA/NKG2D-mediated immunogene therapy of experimental gliomas. Cancer Res. 63, 8996–9006 14695218

[B11] Geller M. A. Miller J. S. (2011). Use of allogeneic NK cells for cancer immunotherapy. Immunotherapy 3, 1445–1459 10.2217/imt.11.131 22091681 PMC3292871

[B12] Huse J. T. Holland E. C. (2010). Targeting brain cancer: advances in the molecular pathology of malignant glioma and medulloblastoma. Nat. Rev. Cancer 10, 319–331 10.1038/nrc2818 20414201

[B13] Kloess S. Huenecke S. Piechulek D. Esser R. Koch J. Brehm C. (2010). IL-2-activated haploidentical NK cells restore NKG2D-mediated NK-cell cytotoxicity in neuroblastoma patients by scavenging of plasma MICA. Eur. J. Immunol. 40, 3255–3267 10.1002/eji.201040568 21061445

[B14] Leary S. E. Olson J. M. (2012). The molecular classification of medulloblastoma: driving the next generation clinical trials. Curr. Opin. Pediatr. 24, 33–39 10.1097/MOP.0b013e32834ec106 22189395 PMC3348176

[B15] Pérez-Martínez A. de Prada Vicente I. Fernández L. González-Vicent M. Valentín J. Martín R. (2012). Natural killer cells can exert a graft-vs-tumor effect in haploidentical stem cell transplantation for pediatric solid tumors. Exp. Hematol. 40, 882–891 10.1016/j.exphem.2012.07.004 22771496

[B16] Pérez-Martínez A. Quintero V. Vicent M. G. Sevilla J. Díaz M. A. Madero L. (2004). High-dose chemotherapy with autologous stem cell rescue as first line of treatment in young children with medulloblastoma and supratentorial primitive neuroectodermal tumors. J. Neurooncol. 67, 101–106 10.1023/B:NEON.0000021774.79094.25 15072454

[B17] Raffaghello L. Nozza P. Morandi F. Camoriano M. Wang X. Garrè M. L. (2007). Expression and functional analysis of human leukocyte antigen class I antigen-processing machinery in medulloblastoma. Cancer Res. 67, 5471–5478 10.1158/0008-5472.CAN-06-4735 17545629

[B18] Rossi A. Caracciolo V. Russo G. Reiss K. Giordano A. (2008). Medulloblastoma: from molecular pathology to therapy. Clin. Cancer Res. 14, 971–976 10.1158/1078-0432.CCR-07-2072 18281528 PMC3222918

[B19] Salmaggi A. Dufour A. Silvani A. Ciusani E. Nespolo A. Boiardi A. (1994). Immunological fluctuations during intrathecal immunotherapy in three patients affected by CNS tumours disseminating via CSF. Int. J. Neurosci. 77, 117–125 10.3109/00207459408986036 7989157

[B20] Sankhla S. K. Nadkarni J. S. Bhagwati S. N. (1996). Adoptive immunotherapy using lymphokine-activated killer (LAK) cells and interleukin-2 for recurrent malignant primary brain tumors. J. Neurooncol. 27, 133–140 10.1007/BF00177476 8699235

[B21] Silvani A. Salmaggi A. Parmiani G. Boiardi A. (1994). Successful adoptive immunotherapy with lymphokine-activated killer cells in the treatment of medulloblastoma disseminated via cerebrospinal fluid: case report. Neurosurgery 34, 1078–1080 10.1227/00006123-199406000-00021 8084395

[B22] Smith C. Santi M. Rajan B. Rushing E. J. Choi M. R. Rood B. R. (2009). A novel role of HLA class I in the pathology of medulloblastoma. J. Transl. Med. 7, 59 10.1186/1479-5876-7-59 19594892 PMC2714836

[B23] Smith C. Santi M. Rushing E. J. Cornelison R. MacDonald T. J. Vukmanovic S. (2011). Characterization of signaling function and expression of HLA class I molecules in medulloblastoma. J. Neurooncol. 103, 197–206 10.1007/s11060-010-0378-3 20811766 PMC3098313

[B24] Sonabend A. M. Ogden A. T. Maier L. M. Anderson D. E. Canoll P. Bruce J. N. (2012). Medulloblasoma: challenges for effective immunotherapy. J. Neurooncol. 108, 1–10 10.1007/s11060-011-0776-1 22173741

[B25] Von Hoff K. Rutkowski S. (2012). Medulloblastoma. Curr. Treat. Options Neurol. 14, 416–426 10.1007/s11940-012-0183-8 22622599

